# Annotation

**Published:** 1953

**Authors:** 


### *Annotation*

### GOOD SENSE ABOUT ANTIBIOTICS

" The United Bristol Hospitals have recently produced an excellent booklet offers a practical guide to the use of Sulphonamides and Antibiotics." So runs a hearted annotation in *The Lancet* (April 25th, 1953).

The merits of this booklet were quickly recognized and the first printings were gone.

A second edition has just been printed and copies may be obtained from the Secr United Bristol Hospitals, Bristol Royal Infirmary, Bristol 2. Price 1s. 6d.

